# Up-regulation of endoplasmic reticulum stress induced genes of the unfolded protein response in the liver of periparturient dairy cows

**DOI:** 10.1186/1746-6148-10-46

**Published:** 2014-02-20

**Authors:** Denise K Gessner, Gloria Schlegel, Robert Ringseis, Frieder J Schwarz, Klaus Eder

**Affiliations:** 1 Institute of Animal Nutrition and Nutrition Physiology, Justus-Liebig-Universität Giessen, Heinrich-Buff-Ring 26-32, Giessen D-35392, Germany; 2 Chair of Animal Nutrition, Center of Life and Food Sciences Weihenstephan, Technische Universität München, Liesel-Beckmann-Strasse 6, Freising, Weihenstephan D-85350, Germany

**Keywords:** Dairy cow, Liver, ER stress, Unfolded protein response

## Abstract

**Background:**

In dairy cows, the periparturient phase is a stressful period, which is commonly associated with strong metabolic adaptations and the development of pathophysiologic conditions and disorders. Some of the symptoms occurring in the liver, such as the development of fatty liver, are similar to those observed under the condition of endoplasmic reticulum (ER) stress. Therefore, we hypothesized, that in the liver of dairy cows ER stress is induced during the periparturient phase, which in turn leads to an induction of the unfolded protein response (UPR). In order to investigate this hypothesis, we determined relative mRNA concentrations of 14 genes of the ER stress-induced UPR in liver biopsy samples of 13 dairy cows at 3 wk antepartum and 1, 5 and 14 wk postpartum.

**Results:**

We found, that the mRNA concentrations of 13 out of the 14 genes involved in the UPR in the liver were significantly increased (1.9 to 4.0 fold) at 1 wk postpartum compared to 3 wk antepartum. From 1 wk postpartum to later lactation, mRNA concentrations of all the genes considered were declining. Moreover, at 1 wk postpartum, mRNA concentration of the spliced variant of XBP1 was increased in comparison to 3 wk antepartum, indicating that splicing of XBP1 – a hallmark of ER stress - was induced following the onset of lactation.

**Conclusion:**

The present study reveals, that ER stress might be induced during the periparturient phase in the liver of dairy cows. We assume that the ER stress-induced UPR might contribute to the pathophysiologic conditions commonly observed in the liver of periparturient cows, such as the development of fatty liver, ketosis or inflammation.

## Background

In dairy cows, the periparturient phase representing the time interval between 3 wk before to 3 wk after parturition is associated with strong metabolic adaptations [1]. Production of milk leads to a strong increase of the energy requirement, which however cannot be met as the food intake capacity is limited. Thus, during early lactation, dairy cows are typically in a negative energy balance, which is compensated by a stimulation of lipolysis in adipose tissue. This leads to strongly increased plasma concentrations of non-esterified fatty acids (NEFA), which are partially taken up into the liver. As the capacity of the liver for β-oxidation during the periparturient phase is insufficient, NEFA are incorporated into triacylglycerols (TAG). As very low-density lipoproteins (VLDL) cannot be produced at sufficient amounts due to a low synthesis of Apo B, TAG are stored in the liver leading to fatty liver syndrome. Moreover, there is commonly a strong stimulation of ketogenesis during early lactation, which can result in ketosis [1-3].

Previously, it has been shown, that increased plasma levels of NEFA, such as observed in dietary or genetic models of obesity or diabetes, are leading to stress of the endoplasmic reticulum (ER) in the liver [4-6]. ER stress is defined as an imbalance between the folding capacity of the ER and the protein load, resulting in the accumulation of unfolded or misfolded proteins in the ER lumen [5]. The disturbance of the ER homeostasis activates an adaptive response known as the unfolded protein response (UPR), which aims to restore ER homeostasis and functions by triggering three kinds of protective cellular responses: (i) up-regulation of ER chaperones, such as immunoglobulin heavy-chain binding protein (BiP), to assist in the refolding of proteins; (ii) attenuation of protein translation, and (iii) degradation of misfolded proteins by the proteasome by a process called ER-associated degradation (ERAD) [7,8]. If ER stress-induced damage is too strong and homeostasis cannot be restored, the UPR can lead to cell death by the induction of apoptosis [9,10]. Sensing of stress in the ER lumen is mediated by three ER stress transducers: inositol requiring 1 (IRE1), PKR-like ER kinase (PERK), and activating transcription factor 6 (ATF6) [5,8]. Under non-stress conditions these transducers are bound to the abundant luminal chaperone BiP preventing them from activating downstream events. When misfolded proteins are accumulating in the ER lumen, BiP dissociates from the stress transducers in order to chaperone the misfolded proteins, which leads to an activation of ER stress transducers and an initiation of the UPR [9,11]. Activation of PERK stimulates the phosphorylation of eukaryotic initiation factor (eIF) 2α, which attenuates protein translation [12]. IRE1 activation causes unconventional splicing of X-box binding protein 1 (XBP1) mRNA and translation into the transcription factor XBP1 [8]. XBP1 up-regulates ER chaperons, components of ERAD and stimulates phospholipid biosynthesis, which leads to an expansion of the ER membrane [5-7]. IRE1 activation moreover leads to an activation of nuclear factor kappa B (NF-κB), a transcription factor involved in inflammation, and an induction of pro-apoptotic genes [5,13]. ATF6 is a transcription factor, which is activated by processing via site 1 and site 2 proteases in the Golgi. The activated ATF6 induces the expression of genes involved in ERAD, lipid biosynthesis, ER expansion and protein folding [5].

Interestingly, activation of the UPR, such as observed in models of obesity or diabetes or induced by application of chemical ER stress inducers, leads to a variety of symptoms in the liver, which are similar to those observed in periparturient dairy cows, such as the development of fatty liver [14-16], an induction of fibroblast growth factor (FGF) 21 [17], an enhancement of the antioxidant and cytoprotective capacity by activation of nuclear factor E2-related factor 2 (Nrf2) [18,19], and an induction of inflammation [20,21].

The fact that periparturient cows have commonly strongly increased plasma concentrations of NEFA and the similarities between the metabolic changes observed in the liver of periparturient cows and those induced by ER stress, prompted us to the hypothesis that the periparturient phase in dairy cows is associated with the development of ER stress in the liver. In order to investigate this hypothesis, we determined mRNA concentrations of several important components of the three branches of the UPR operating as chaperons, foldases, components of ERAD or inducers of apoptosis (Table 1) in liver biopsy samples of dairy cows during the periparurient phase. mRNA concentrations of these genes have been proposed as reliable markers of ER stress [22-25].

**Table 1:** Functions of the ER stress-induced genes considered in this study

**Component**^**1**^	**Function**	**Reference**
*ATF4*	Indicator of PERK activation, transcription factor which contributes to the transcriptional activation of chaperones and foldases	[5]
*BAK1*	ER stress induced pro-apoptotic gene of the BCL-2 family proteins	[10]
*BAX*	ER stress induced pro-apoptotic gene of the BCL-2 family proteins	[26]
*HSPA5* (encoding BiP)	Chaperon, master regulator of UPR	[5,27]
*CASP3, CASP8, CASP9* (encoding caspases 3, 8, 9)	ER stress induced members of a family of cysteine proteases which are critical mediators of apoptosis	[9,13,27,28]
*DDIT3* (encoding CHOP)	Non ER localised transcription factor, induced by ER stress through PERK and ATF6, mediates ER stress induced apoptosis	[29]
*EDEM1*	ER stress induced target of IRE, component of the ER-associated degradation system (ERAD)	[29,30]
*HERPUD1* (encoding HERP)	ER stress induced target of ATF6, a resident membrane protein involved in the ERAD complex	[31]
*PDIA4*	ER stress induced target of PERK, one of the most important ER resident protein folding enzymes with chaperone activity in preventing the aggregation of unfolded substrates	[25]
*DNAJC3* (encoding P58^IPK^)	ER stress induced target of IRE, a molecular chaperone	[32]
*WARS*	ER stress induced target of PERK, ATF4 target gene, aminoacyl-tRNA synthetase involved in protein synthesis	[33]
*XBP1*	ER stress induced target of IRE, transcription factor which induces the transcriptional activation of chaperones	[5]

^1^*ATF4* = activating transcription factor 4; *BAK1* = BCL2-antagonist/killer 1; *BAX* = BCL2-associated X protein; *CASP* = apoptosis-related cysteine peptidase; *DDIT3* = DNA-damage-inducible transcript 3; DNAJC3 = DnaJ (Hsp40) homolog, subfamily C, member 3; *EDEM1* = ER degradation enhancer, mannosidase alpha-like 1; *HERPUD1* = homocysteine-inducible, endoplasmic reticulum stress-inducible, ubiquitin-like domain member 1; *HSPA5* = heat shock 70 kDa protein 5; *PDIA4* = protein disulfide isomerase family A, member 4; *WARS* = tryptophanyl-tRNA synthetase; *XBP1* = X-box binding protein 1.

## Results

Performance parameters of the cows used in this experiment have recently been reported [34]. Milk yield of the cows, in average from wk 1 to wk 14 was approximately 33 kg/d, average feed intake was 18.5 kg DM/d. The onset of lactation led to a strong negative energy balance of about -65 MJ NEL/d in wk 1. At 14 wk postpartum, the cows had a slightly positive energy balance. Plasma NEFA concentrations showed their peak values at 1 wk postpartum while plasma β-hydroxybutyric acid (BHBA) concentrations were highest at 1 wk and 5 wk postpartum. Liver TAG concentrations were highest at 5 wk postpartum [34]. The cows considered in this study, moreover, showed increased mRNA concentrations of tumor necrosis factor α and acute phase proteins (C-reactive protein, haptoglobin, serum amyloid A) being indicative of a pro-inflammatory condition and increased mRNA concentrations of various Nrf2 target genes and of FGF21 in the liver at 1 wk postpartum [35,36].

**Figure 1:**
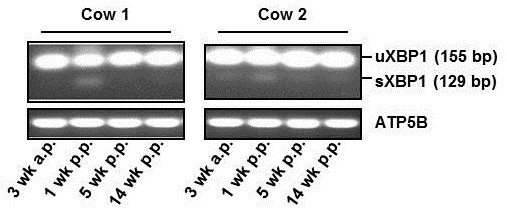
**Activation of XBP1 by unconventional splicing following the onset of lactation.** Representative images from two cows demonstrating the unspliced (155 bp) and the spliced (129 bp) XBP1 mRNA as determined by conventional RT-PCR. The ATP5B mRNA was determined as reference gene.

To detect an activation of the UPR due to ER stress, we determined mRNA concentrations of BiP (encoded by *HSPA5*) and 13 downstream genes of the three ER stress sensors by qRT-PCR and the mRNA concentration of the spliced variant of XBP1 (sXBP1) by standard RT-PCR. The mRNA concentrations of all the UPR target genes, with the only exception of *WARS*, were elevated from 3 wk antepartum to 1 wk postpartum (Table 2). Moreover, at 1 wk postpartum, mRNA concentration of sXBP1 was increased in comparison to 3 wk antepartum, indicating that splicing of XBP1 was induced following the onset of lactation (Figure 1). From 1 wk postpartum to later lactation, mRNA concentrations of all the genes involved in the UPR were declining. However, relative mRNA concentrations of some genes considered (*ATF4, CASP3, EDEM1, WARS, XBP1*) were also significantly increased at 5 wk postpartum in comparison to 3 wk antepartum (Table 2). The mRNA concentrations of most of the genes considered were not different between 14 wk postpartum and 3 wk antepartum, while mRNA concentrations of ATF4 and the 114 bp unspliced XBP1 were higher at 14 wk postpartum than at 3 wk antepartum.

**Table 2:** **Relative mRNA concentrations of ER stress-induced genes in the liver of Holstein cows at 3 wk antepartum and 1, 5 and 14 wk postpartum**^**1**^

**Gene**^**2**^	**3 wk antepartum (n = 13)**	**1 wk postpartum (n = 13)**	**5 wk postpartum (n = 13)**	**14 wk postpartum (n = 13)**
*ATF4*	1.00 ± 0.25^a^	3.14 ± 0.25^c^	2.08 ± 0.26^b^	1.65 ± 0.24^b^
*BAK1*	1.00 ± 0.29^a^	2.10 ± 0.30^b^	1.56 ± 0.29^ab^	1.59 ± 0.28^ab^
*BAX*	1.00 ± 0.36^a^	3.02 ± 0.39^b^	1.98 ± 0.35^ab^	1.52 ± 0.35^a^
*CASP3*	1.00 ± 0.31^a^	2.54 ± 0.27^b^	2.07 ± 0.26^b^	1.71 ± 0.26^ab^
*CASP8*	1.00 ± 0.25^a^	2.48 ± 0.24^b^	1.88 ± 0.23^ab^	1.73 ± 0.23^ab^
*CASP9*	1.00 ± 0.37^a^	2.45 ± 0.35^b^	1.89 ± 0.34^ab^	1.84 ± 0.36^ab^
*DDIT3*	1.00 ± 0.17^a^	1.86 ± 0.17^b^	1.58 ± 0.17^ab^	1.41 ± 0.17^ab^
*DNAJC3*	1.00 ± 0.15^a^	1.89 ± 0.17^b^	1.49 ± 0.17^ab^	1.43 ± 0.15^ab^
*EDEM1*	1.00 ± 0.21^a^	1.70 ± 0.21^b^	1.69 ± 0.20^b^	1.25 ± 0.22^ab^
*HERPUD1*	1.00 ± 0.45^a^	3.96 ± 0.37^b^	2.27 ± 0.43^a^	1.66 ± 0.38^a^
*HSPA5*	1.00 ± 0.29^a^	2.74 ± 0.27^b^	1.64 ± 0.26^a^	1.41 ± 0.24^a^
*PDIA4*	1.00 ± 0.25^a^	1.92 ± 0.28^b^	1.34 ± 0.24^ab^	1.23 ± 0.25^ab^
*WARS*	1.00 ± 0.35^a^	2.03 ± 0.31^ab^	2.31 ± 0.29^b^	1.93 ± 0.30^ab^
*XBP1 (unspliced)*	1.00 ± 0.25^a^	3.14 ± 0.25^c^	2.08 ± 0.26^b^	1.65 ± 0.24^b^

^1^mRNA concentrations of genes are expressed relative to the mRNA abundance at 3 wk antepartum.

^2^Abbreviations of gene names: see Table 1.

Values are means ± SEM.

^a,b,c^Means without common superscripts differ significantly (*P <* 0.05).

We also determined the mRNA concentrations of genes associated with DNA damage, DNA repair, and cell cycle (*ATM, BRCA1, HSBP1, HSPA8, MSH2, RPS9, XRCC5*) (Table 3). Those genes are not regulated by ER stress, and thus can be used to evaluate whether ER stress is specifically induced. However, we observed that the mRNA concentrations of most of these genes (with the exception of *BRCA1*) showed the same expression pattern as the abovementioned ER stress-regulated genes (increase from 3 wk antepartum to 1 wk postpartum and decrease from 1 wk to 14 wk postpartum) indicating that genes involved in DNA damage, DNA repair, and cell cycle are similarly regulated in the liver of cows during lactation as ER stress-regulated genes.

**Table 3:** **Relative mRNA concentrations of genes associated with DNA damage, DNA repair and cell cycle in the liver of Holstein cows at 3 wk antepartum and 1, 5 and 14 wk postpartum**^**1**^

**Gene**^**2**^	**3 wk antepartum (n = 13)**	**1 wk postpartum (n = 13)**	**5 wk postpartum (n = 13)**	**14 wk postpartum (n = 13)**
*ATM*	1.00 ± 0.21^a^	2.29 ± 0.21^b^	1.94 ± 0.35^ab^	1.81 ± 0.26^ab^
*BRCA1*	1.00 ± 0.22	1.71 ± 0.27	1.59 ± 0.20	1.33 ± 0.23
*HSBP1*	1.00 ± 0.23^a^	3.04 ± 0.42^b^	2.14 ± 0.24^ab^	1.65 ± 0.34^a^
*HSPA8*	1.00 ± 0.24^a^	2.01 ± 0.24^b^	1.78 ± 0.23^ab^	1.22 ± 0.27^a^
*MSH2*	1.00 ± 0.20^a^	4.04 ± 0.61^b^	1.81 ± 0.22^a^	1.41 ± 0.23^a^
*RPS9*	1.00 ± 0.20^a^	3.68 ± 0.28^c^	2.23 ± 0.29^b^	1.64 ± 0.31^ab^
*XRCC5*	1.00 ± 0.21^a^	3.69 ± 0.54^c^	2.35 ± 0.27^b^	1.43 ± 0.26^a^

^1^mRNA concentrations of genes are expressed relative to the mRNA abundance at 3 wk antepartum.

^2^*ATM* = ataxia telangiectasia mutated; BRCA1 = breast cancer 1, early onset; HSBP1 = heat shock factor binding protein 1; *HSPA8* = heat shock 70 kDa protein 8; *MSH2* = mutS homolog 2; RPS9 = ribosomal protein S9; *XRCC5* = X-ray repair cross-complementing protein 5-like.

Values are means ± SEM.

^a,b,c^Means without common superscripts differ significantly (*P <* 0.05).

## Discussion

**Figure 2:**
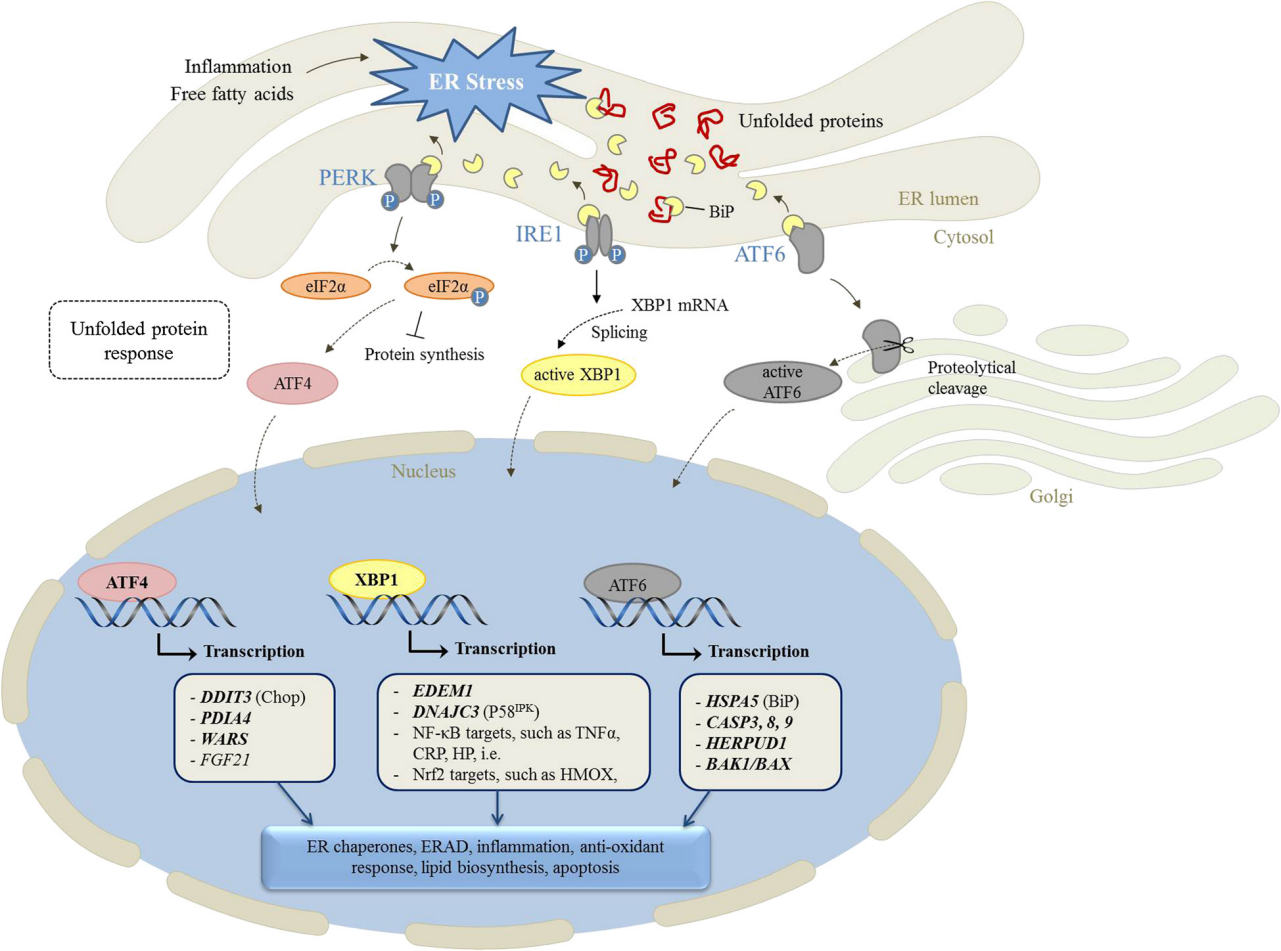
**Endoplasmic reticulum (ER) stress signal transduction (Modification of [**[21]**]).** Genes considered in the liver of periparturient dairy cows in this study are shown in bold. ER stress in early lactating cows might be induced either by inflammation and/or high concentrations of NEFA deriving from plasma due to strong lipolysis in adipose tissue. In response to ER stress, the three ER stress sensors PKR-like ER kinase (PERK), inositol requiring 1 (IRE1), and activating transcription factor 6 (ATF6) are activated by dissociation from BiP. In turn, genes of the UPR including those considered in this study (*ATF4, DDIT3, PDIA4, WARS, EDEM1, DNAJC3, HSPA5, CASP3, CASP8, CASP9, HERPUD1, BAK1, BAX*) are up-regulated. Increased mRNA concentrations of FGF21 and target genes of NF-κB and Nrf2, which are also induced by ER stress in the liver of periparturient cows, have been recently reported [34,35,39,40].

In the present study, we observed that BiP, a chaperone which is considered as the master regulator of the UPR [5], and several downstream genes of the three ER stress transducers (IRE1, PERK, ATF6) are up-regulated in the liver of dairy cows at early lactation (Figure 2). It has been assumed that mRNA concentrations of ER chaperons, ERAD components, such as BiP, HERP, WARS, PDIA4, P58^IPK^, EDEM1, XBP1, and ATF4, or genes, that are involved in the induction of apoptosis, such as Chop or caspases, are reliable markers of ER stress [22-24,27,37]. Thus, the present study strongly suggests the presence of ER stress in the liver of dairy cows during early lactation, which was associated with induction of the UPR. This suggestion is supported by the present finding, that the mRNA concentration of sXBP1 in the liver was increased at 1 wk postpartum, indicative of an increased XBP1 splicing, which is considered a hallmark of ER stress [8]. The observed activation of XBP1 by unconventional splicing of XBP1 mRNA 1 wk postpartum agrees with a recent study of Loor [38] who found an up-regulation of 39 target genes of XBP1 in the liver of dairy cows during the transition from late pregnancy to lactation by transcriptome analysis.

From the present study, the metabolic reasons for the production of ER stress during early lactation cannot be explained. However, it has been shown that an increased load of the liver with fatty acids induces ER stress, with saturated fatty acids being more deleterious in this respect than unsaturated fatty acids [6,16,37]. Saturated fatty acids are less readily converted into TAG than unsaturated fatty acids, and thus travel to the ER in the free form, where they may disrupt ER morphology and function [16]. Thus, it is likely that the elevated concentrations of NEFA, consisting mainly of saturated fatty acids and oleic acid [41], in the blood of the cows in early lactation could contribute to the induction of ER stress in the liver. ER stress induction in the liver of cows during early lactation could also be due to the occurrence of a pro-inflammatory condition. Periparturient cows exert commonly an inflammation-like condition in the liver, induced by various events such as injuries and trauma during calving stress, mammary gland oedema, uterus involution, infectious or metabolic diseases, parasites or endotoxins from the gut [42-44]. A pro-inflammatory condition during early lactation has also been observed in the cows considered in this study [35]. As inflammation strongly induces ER stress [18], activation of the UPR in the liver of the cows could have been triggered by the pro-inflammatory condition. The finding that the expression of UPR target genes was declining from 1 wk postpartum to later lactation supports the hypothesis that ER stress in early lactation was caused mainly by high plasma levels of NEFA and the inflammatory condition. However, the finding that some of the UPR target genes remained up-regulated even at 14 wk postpartum – when the cows were already in a positive energy balance – suggests that a certain degree of ER stress could also be induced by the high metabolic activity of the liver during lactation, without being burdened by high NEFA concentrations or inflammation.

Many biochemical alterations in the liver induced by UPR under pathophysiologic conditions such as obesity, diabetes or chronic inflammation are similar to those observed in the liver of periparturient dairy cows, with fatty liver development being one important example. ER stress-induced fatty liver is caused by an increased expression of genes involved in lipogenesis, a reduced expression of genes involved in fatty acid oxidation and lipolysis, and an impairment of the production of VLDL required for the export of TAG from the liver [14,45-47]. As these biochemical alterations leading to fatty liver are very similar to those observed in the liver of periparturient dairy cows [1,2,48], it is likely that the ER stress-induced UPR is involved in the development of fatty liver in periparturient cows. Other similarities between various biochemical alterations induced by ER stress and those observed in the liver of periparturient dairy cows are the induction of inflammation, an activation of Nrf2 pathway and an up-regulation of FGF21. As mentioned above, inflammation can directly induce ER stress. However, ER stress and the concomitant UPR also enhance the inflammatory process [18], suggesting that ER stress could contribute to the induction of the pro-inflammatory condition in the liver of periparturient cows. Nrf2 is a transcription factor which regulates the transcription of a great number of genes with antioxidative and cytoprotective functions [49,50]. Activation of this transcription factor during the periparturient phase in the liver of the cows has been considered as a compensatory means to protect the liver against the deleterious effects of pro-inflammatory cytokines and reactive oxygen species (ROS) [36]. The fact that ER stress also causes an activation of Nrf2, probably as a means to counteract oxidative stress provoked under ER stress conditions [18,19] suggests that the observed activation of Nrf2 in the liver of dairy cows at early lactation might also be caused by the UPR.

FGF21 is a hormonal regulator which stimulates hepatic lipid oxidation, ketogenesis and gluconeogenesis during energy deprivation [51-53]. Recently, it has been found that the expression of FGF21 in the liver and plasma levels of FGF21 are increased in dairy cows during the periparturient phase, and that there is even a relationship between hepatic TAG content and plasma FGF21 concentration in dairy cows [34,39,40]. More recently, it has been observed that FGF21 is directly induced by ER stress, mediated by an activation of the PERK cascade [17]. Thus, it is likely that the up-regulation of FGF21 in the liver of periparturient cows is mediated by an ER stress-induced UPR. The fact that FGF21 stimulates ketogenesis indicates that ER stress present in the liver of periparturient cows might enhance the development of ketosis via an up-regulation of FGF21.

Based on the similarities between various biochemical alterations induced by ER stress and those observed in the liver of dairy cows during the periparturient phase, it is probable that the induction of ER stress and the concomitant UPR contribute to pathophysiologic conditions during this phase, such as the development of fatty liver, ketosis, and hepatic inflammation. The existence of ER stress in the liver of dairy cows, moreover, might be of relevance for glucose homeostasis as it has been shown that ER stress impairs gluconeogenesis [54,55], a pathway, which is of extraordinary relevance in high yielding dairy cows.

It should be noted that the induction of ER stress gene network expression in dairy cows has been also observed in the mammary gland during the transition from pregnancy to lactation [56]. The physiological relevance of that pathway, however, might be different between liver and mammary gland. While ER stress induced UPR in the liver might be regarded as a means to maintain ER homeostasis and liver function, ER stress signalling in the mammary gland might be involved in mammary lipogenesis and milk protein synthesis [56].

## Conclusion

In overall, the present study reveals the existence of ER stress in the liver of dairy cows during early lactation. Since ER stress causes many biochemical adaptations and symptoms similar to those observed in the liver of periparturient cows, such as the development of fatty liver, ketosis or inflammation, it is assumed that the ER stress-induced UPR might contribute to the pathophysiologic conditions commonly observed in the liver of periparturient cows.

## Methods

### Animals

For this investigation, liver biopsy samples from a recently performed trial with dairy cows, which has been described in detail [34], were used. All procedures for this trial were approved by the Bavarian state animal care and use committee. The trial included twenty Holstein cows (four primi- and sixteen multiparous, 2.7 ± 1.1 parities, mean ± SD) as experimental animals with an experimental period from 3 wk antepartum until 14 wk postpartum. The animals were housed in a freestall-barn. They received a partial mixed ration (PMR) for *ad libitum* intake of basic feed with separate and limited intake of concentrate. PMR consisted (dry matter, DM, basis) of 33.7% grass silage, 44.9% maize silage, 6.4% hay, and 14.9% concentrate. The concentrate was individually allocated at four computer-operated feeding stations with an automatic feeding program (DeLaval Alpro, Glinde, Germany). It was composed of 24.8% grain maize, 21.8% wheat, 20.1% soybean meal, 15.2% dried sugar beet pulp with molasses, 14.9% barley and 3.2% vitamin-mineral premix including limestone (DM basis). The allocation of the concentrate was increased from 1.2 to 8.0 kg of DM/d during the first 42 d of lactation, and thereafter, it was dependent on the milk performance of the individual cow. Liver biopsies were taken from the right liver lobe (*Lobus hepatis dexter*) at 3 wk antepartum, and 1, 5 and 14 wk postpartum before feeding between 0700 and 0900 h [34]. For this study, samples of 13 cows were available.

### Quantitative and standard RT-PCR

Total RNA isolation from liver biopsies, cDNA synthesis and quantitative real-time polymerase chain reaction (qPCR) were carried out as described recently in detail [34]. Expression values of the genes investigated were normalised using the GeNorm normalisation factor [57]. Procedure of normalisation and average expression stability ranking of the six potential reference genes in liver of cows were also performed as described recently [34]. The characteristics of gene-specific primers are shown in Table 4. After normalisation of gene expression data using the calculated GeNorm normalisation factor, means and SEM were calculated from normalised expression data for samples of the same treatment group. The mean of 3 wk antepartum was set to 1 and relative expression ratios of 1, 5 and 14 wk postpartum are expressed as fold changes compared to 3 wk antepartum. To determine the expression of spliced and unspliced XBP1, the PCR run was stopped within the linear range of amplification. Subsequently, the PCR products were separated using 2% agarose gel electrophoresis stained with GelRedTM nucleic acid gel stain (Biotium, California, USA), visualized under UV light, and digitalized with a digital camera (SynGene, Cambridge, England).

**Table 4:** Characteristics of gene specific primers used for qPCR

**Gene**^**1**^	**Forward primer (from 5′ to 3′) Reverse primer (from 5′ to 3′)**	**PCR product size (bp)**	**NCBI GenBank**
Reference genes			
*ATP5B*	GGACTCAGCCCTTCAGCGCC GCCTGGTCTCCCTGCCTTGC	229	NM_175796.2
*PPIA*	GGCAAATGCTGGCCCCAACACA AGTACCACGTGCTTGCCATCCA	87	NM_178320.2
*RPL12*	CACCAGCCGCCTCCACCATG CGACTTCCCCACCGGTGCAC	84	NM_205797.1
Target genes			
*ATF4*	TGGTCTCAGACAACAGCAAG AGCTCATCTGGCATGGTTTC	130	NM_001034342.2
*ATM*	GTGTTGAGGCACTTTGTGATGC GTTTGATAATGGGCTGGTCTGC	111	NM_001205935.1
*BAK1*	TACTTCACCAAGATCGCGTC ACGATGGCTACGCTCTTGAT	254	NM_001077918.1
*BAX*	TCTGACGGCAACTTCAACTG ATGGTCACTGTCTGCCATGT	224	NM_173894.1
*BRCA1*	TGCCGAGACAAGATCAAGAGG TAATTTCAGTGCAGAGGCTGAGG	149	NM_178573.1
*CASP3*	CCGAGGCACAGAACTGGACTG TCGCCAGGAAAAGTAACCAGGTG	133	NM_001077840.1
*CASP8*	TACCAGCGAGGAGGAGATGAAG CATCCAGCTTACATTTGGCAATC	164	NM_001045970.2
*CASP9*	AAACAGGATGACCCATCAAAGC ATTCAGGACATAGGCCAGATCG	203	NM_001205504.1
*DDIT3*	AGTCACTGCCTTTCTCCTTC TCTTCCTCCTTGTTTCCAGG	133	NM_001078163.1
*DNAJC3*	GTACGAAGGTGCTGAATGTG ATCAGGGTCACCATCTACTG	133	NM_174756.3
*EDEM1*	CCCCTACCCTCGGGTGAATCT GTGGAATCCCCCAGCAGTCG	126	NM_001103092.2
*HERPUD1*	CCGTGTTTCTCAGTATCCTC TCTTGATTCACAGCCTCCTG	169	NM_001102265.2
*HSBP1*	CGCGAACAAACGGAAGTATAGG CAGGTCATCAATGCGACTGC	207	NM_001113316.2
*HSPA5*	CAAGTTGATGTTGGAGGTGG AAGCCTCAGCAGTTTCCTTC	94	NM_001075148.1
*HSPA8*	AACGTGCTGATCTTTGATTTAGGG TTCTCCACCCAAGTGAGTATCTCC	114	NM_174345.4
*MSH2*	AACAGAAAGCCCTGGAGTTGG TTATTCTTTGCGATGACCTCAGC	226	NM_001034584.1
*PDIA4*	AGGTTTGACGTGAGTGGCTA CATCGAAGTTGTCCTTGGTC	175	NM_001045879.2
*RPS9*	GTGAGGTCTGGAGGGTCAAA GGGCATTACCTTCGAACAGA	108	NM_001101152.2
*WARS*	AAGCAGACGAGGACTTTGTG TTCGGTTTACCAGCTCCTTG	123	NM_174218.1
*XBP1 unspliced*	GTTGAGACAGCGGTTGGGAATG CGTAGTCTGAGTGCTGCGGAC	114	NM_001034727.3
*XBP1 spliced & unspliced*	TGACTGAAGAGGAAGCAGAG CAATGCCATCAGAGTCCATG	129/155	NM_001271737.1
*XRCC5*	GTTTCAGTGTCTGCTTCACAGAGC TTCTTGATGACTTCCGTCAGAGG	165	NM_001102141.1

^1^*ATP5B* = ATP synthase; *PPIA* = peptidylprolyl isomerase A; *RPL12* = ribosomal protein L12; other abbreviations of gene names are explained in footnotes of Tables 1 and 3.

### Statistical analysis

Data were statistically evaluated by using the SAS procedure PROC MIXED (version 9.2, SAS Institute Inc., Cary, NC, USA) with week of sampling (-3, +1, +5, +14 wk) and parity of the cow (primi- vs. multiparous) as fixed effects, and individual animal as random effect. For significant t values of the factor week, means were compared by the Bonferroni *t*-test, and differences between means were considered significant for *P <* 0.05.

## Competing interests

The authors declare that they have no competing interests.

## Authors’ contributions

DKG: Participated in the design of the study, performed the PCR analyses, performed the statistical analyses, and wrote the manuscript. GS: conducted the animal experiment. FJS: supervised the animal experiment; RR: supervised PCR analyses. KE: conceived of the study, and participated in its design and coordination and helped to draft the manuscript. All authors read and approved the final manuscript.
